# Characteristics and outcomes of men presenting with complications of metastatic prostate cancer

**DOI:** 10.1111/bju.70179

**Published:** 2026-02-17

**Authors:** Arjun Nathan, Matthew G. Parry, Adrian Cook, Emily Mayne, Joanna Dodkins, Marina Parry, Raghav Varma, Ajay Aggarwal, Jan van der Meulen, James S. A. Green, Alison Tree, Noel Clarke, Thomas E. Cowling

**Affiliations:** ^1^ Department of Health Services Research and Policy London School of Hygiene and Tropical Medicine London UK; ^2^ National Cancer Audit Collaborating Centre, Clinical Effectiveness Unit Royal College of Surgeons of England London UK; ^3^ Department of Surgery and Interventional Science University College London London UK; ^4^ Guy's Cancer Centre Guy's and St Thomas’ NHS Foundation Trust London UK; ^5^ Department of Urology Barts Health NHS Foundation Trust London UK; ^6^ Department of Urology The Royal Marsden NHS Foundation Trust and The Institute of Cancer Research London UK; ^7^ Department of Urology The Christie NHS Foundation Trust Manchester UK; ^8^ Division of Cancer Sciences The University of Manchester Manchester UK

**Keywords:** prostate cancer, metastatic, complication, malignant ureteric obstruction, skeletal bone

## Abstract

**Objectives:**

To describe the incidence, characteristics and mortality of men who present with complications of metastatic prostate cancer, a previously under‐reported population. We investigate men who present with and without malignant ureteric obstruction (MUO) and skeletal‐related events (SREs), collectively termed ‘metastatic‐related events’ (MREs).

**Patients and Methods:**

We used the English Cancer Registry linked to hospital administrative data to identify men diagnosed with metastatic prostate cancer between January 2015 and December 2022. Poisson regression models estimated adjusted relative risks (aRRs) of presenting with MREs. The cumulative incidences of overall and prostate cancer‐specific death were estimated for each MRE subgroup (metastatic without MRE, MUO, SRE, and MUO and SRE in combination).

**Results:**

Of 48 171 men diagnosed with primary metastatic disease, 4272 (8.9%) presented with MREs. Of these men, 2453 (57.4%) had MUO, 1738 (40.7%) had a SRE, and 81 (1.9%) had both. Men aged ≥80 years had the highest risk (9.8% [1604/16452]) of presenting with MREs. Men aged 70–79 years (8.0% [1470/18397]) (aRR 0.82, 95% confidence interval [CI] 0.77–0.88) and men aged 60–69 years (8.9% [916/10297]) (aRR 0.91, 95%CI 0.84–0.98) had lower risks. Men from the most deprived neighbourhoods (9.3% [706/7609]) (aRR 1.27, 95% CI 1.15–1.40) had greater risks of presenting with MREs than those from the least deprived neighbourhoods (8.0% [868/10865]). The proportion of men presenting with MREs varied across geographical regions, ranging from 4.6% (288/6233) to 11.7% (461/3951). The 5‐year overall mortality for men presenting without MREs was 57.8% (95% CI 57.2–58.4%), compared to 77.1% (95% CI 74.9–79.2%) with MUO, 66.8% (95% CI 64.1–69.4%) with a SRE and 84.4% (95% CI 74.1–94.7%) with both.

**Conclusions:**

The risk of presenting with metastatic prostate cancer and MREs varies according to age, socioeconomic deprivation, and residential region. These men have poorer survival outcomes than men diagnosed without MREs at diagnosis.

**Abbreviations**
aRR: adjusted relative risk
COVID‐19: coronavirus disease 2019
HES: Hospital Episode Statistics
ICD‐10: International Classification of Diseases, 10th edition
MRE: metastatic‐related event
MUO: malignant ureteric obstruction
NCRD: National Cancer Registration Dataset
ONS: Office for National Statistics
SRE: skeletal‐related event

## Introduction

Across high‐income countries, approximately one in five men diagnosed with prostate cancer present with metastatic disease [1]. The reported median overall survival of these men ranges from 3 to 6 years [2, 3].

Prostate cancer metastases may cause various complications. Most commonly, bone deposits may lead to fractures or spinal cord compression, known as skeletal‐related events (SREs). Additionally, metastases or local infiltration of prostate cancer in the bladder may obstruct the urinary tract, causing malignant ureteric obstruction (MUO). Collectively, we refer to MUO and SREs as ‘metastatic‐related events’ (MREs) [4]. A MRE often requires urgent surgical intervention in addition to anti‐cancer treatment. The acute and chronic effects of the MRE are frequently debilitating, requiring repeated medical intervention, with a substantial reduction in quality of life [5, 6].

Men may develop MREs after a diagnosis of prostate cancer, or they may present for the first time with a MRE as the cardinal symptom of an underlying undiagnosed prostate cancer. Epidemiological data related to MREs are limited, and there is little published evidence about their incidence or their impact on prognosis when they are present at the time of prostate cancer diagnosis.

Using linked National Cancer Registry and English NHS hospital records, we describe a new population of men who present with a MRE at the time of metastatic prostate cancer diagnosis. We aimed to identify groups at greatest risk who may benefit most from earlier detection, and to estimate their survival outcomes, to inform prognosis.

## Patients and Methods

### Study Population

We included men aged ≥18 years presenting between 1 January 2015 and 31 December 2022 with metastatic prostate cancer at first diagnosis. These men were identified in the English National Cancer Registration Dataset (NCRD) using the International Classification of Diseases, 10th edition (ICD‐10) code for prostate cancer ‘C61’ [7, 8]. Incomplete TNM data were imputed using two validated clinical assumptions. First, men with a recorded N‐status but missing M‐status, were assumed to be M0, as they were likely to have been investigated for metastases, with none found. Second, patients with T1 or T2 disease were also classified as non‐metastatic due to the low likelihood of metastatic disease [9].

### Metastatic‐Related Events

A MRE was defined as either MUO (ureteric obstruction due to prostate cancer with associated hydronephrosis) or a SRE (pathological bone fracture, spinal cord compression, bone surgery or bone pain requiring palliative radiotherapy as a result of prostate cancer). Validated coding frameworks were used to identify MUO and SREs based on ICD‐10 diagnosis codes and Office of Population Censuses and Surveys (OPCS‐4) procedure codes (Data S1 Appendix 1) from Hospital Episode Statistics (HES) records of admitted patients. The HES dataset includes routinely collected administrative hospital data of all care episodes in the English NHS [10, 11]. The National Radiotherapy Dataset (RTDS) identified patients undergoing palliative radiotherapy [12].

A MRE at presentation was defined as a MRE occurring within 30 days before or after the date of the recorded prostate cancer diagnosis. MREs occurring before diagnosis were included to allow sufficient time for diagnostic investigations following initial presentation. MREs occurring after the date of diagnosis were included to account for inconsistencies in the recording of the date of the prostate cancer diagnosis. The 30‐day interval at either side of the date of diagnosis to define an MRE ‘at presentation’ was chosen after visual inspection of the distribution of the observed time differences of the MRE in HES and the date of diagnosis in the NCRD (Data S1 Appendix 2).

### Overall Mortality and Prostate Cancer‐Specific Mortality

Overall mortality was derived from the Office for National Statistics (ONS) death certificates [13]. Prostate cancer‐specific mortality was defined using the ICD‐10 code ‘C61’ as the cause leading to death [14]. The ONS death records were available until 31 July 2024.

### Patient Characteristics

The NCRD data were used to define the year of diagnosis, patient age at diagnosis, ethnicity, socioeconomic deprivation, English NHS region of residence, PSA, Gleason score and TNM stage [15]. Age at diagnosis was grouped into four categories (18–59, 60–69, 70–79 and ≥80 years). Ethnicity was defined as per the ONS five‐group classification: ‘White’, ‘Black’, ‘Asian’ (including Chinese and Indian), ‘Mixed’, ‘Other’, and missing [16]. Missing ethnicity data in the NCRD were populated using HES records where available. Socioeconomic deprivation levels were defined as quintiles of the national distribution of the Index of Multiple Deprivation (IMD) for a patient's neighbourhood, defined as the Lower‐layer Super Output Area of residence, comprising between 1000 and 3000 people and between 400 and 1200 households [17]. An established ‘routes to diagnosis’ methodology developed by NHS England and Public Health England using NCRD and HES data was used to identify patients who were diagnosed with prostate cancer due to an emergency presentation [18].

### Statistical Methods

A MRE at presentation was described according to year of diagnosis and patient characteristics. We used modified Poisson regression models with robust standard errors to estimate unadjusted and adjusted relative risks (aRR) with 95% CIs, adjusting for age, ethnicity, socioeconomic deprivation, and NHS region of residence [19]. Missing patient characteristics were handled using the missing indicator method, where an extra ‘missing’ category was created for each patient characteristic [20]. The Pearson chi‐square test was used to test the association between patient characteristics and risk of being diagnosed with a MRE at presentation. The Wald test was used to test whether the aRRs were equal to 1. The *P* values were two‐sided, and a *P* < 0.05 was considered to indicate a statistically significant difference.

Cumulative incidence estimates were plotted for overall mortality and prostate cancer‐specific mortality. When estimating prostate cancer‐specific mortality, we defined death not from prostate cancer as a competing event [21]. The log‐rank test was used to test the differences in overall mortality and Gray's test was used to test differences in prostate cancer‐specific mortality [22]. Data analysis was undertaken using Stata, version 17 (StataCorp LLC, College Station, TX, USA).

## Results

**Fig. 1:**
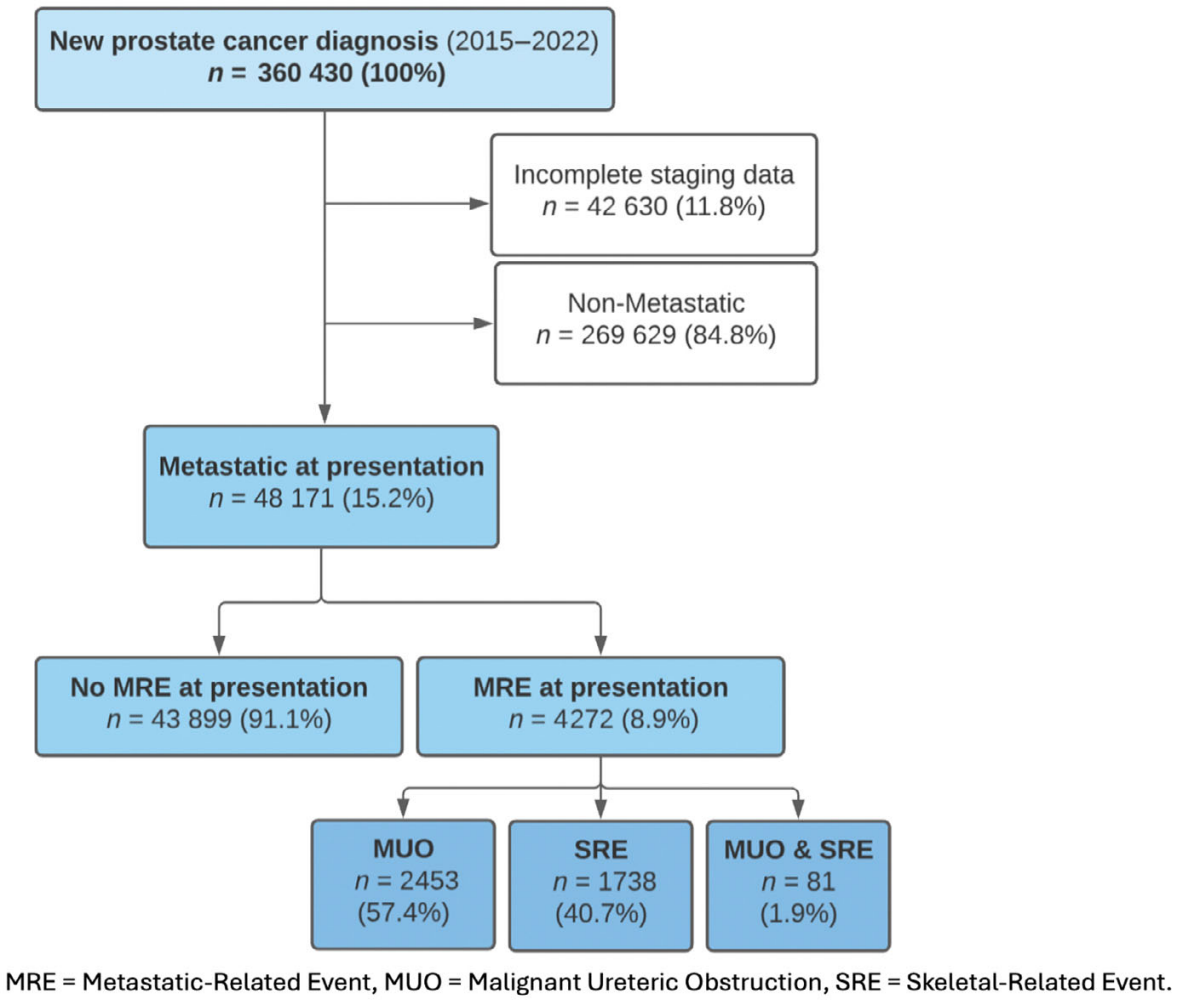
Study population.

Between 1 January 2015 and 31 December 2022, 360 430 men were newly diagnosed with prostate cancer in England. A total of 42 630 (11.8%) men had incomplete TNM staging data and were excluded from the analyses. Of the remaining men, 48 171 (15.2%) had metastatic disease at diagnosis and were included in the analysis as our study population of interest. Of these men, 4272 (8.9%) had a MRE at the time of prostate cancer diagnosis, of whom 2453 (57.4%) had MUO alone and 1738 (40.7%) had a SRE alone; 81 (1.9%) men had both MUO and a SRE. The most common type of SRE was palliative radiotherapy (65.2% [1134/1738]), of which 615 of the 1134 patients had another SRE present. Spinal cord compression (49.0%, [852/1738]) was the next most common SRE subtype, followed by pathological fracture with or without surgical fixation (20.5% [356/1738]). The study population is outlined in Fig. 1.

During the 8‐year study period, the annual number of men diagnosed with primary metastatic disease remained relatively stable (Table 1). The percentage of men with a MRE at presentation ranged between 7.9% and 8.9%, except in 2020 and 2021, during the coronavirus disease 2019 (COVID‐19) lockdown [23], when it increased to 11.0% and 10.0%, respectively.

**Table 1:** Number of men diagnosed annually with primary metastatic prostate cancer and the proportion of those presenting with MUO and/or a SRE, defined collectively as a MRE.

Year	Primary metastatic prostate cancer, *N*	Presenting with a MRE, *n* (%)			
		All MRE	MUO	SRE	MUO and SRE
**Total**	48 171	4272 (8.9)	2453 (5.1)	1738 (3.6)	8 (0.2)
2015	5869	462 (7.9)	268 (4.6)	188 (3.2)	6 (0.1)
2016	6133	522 (8.5)	277 (4.5)	235 (3.8)	10 (0.2)
2017	6350	528 (8.3)	272 (4.3)	247 (3.9)	9 (0.1)
2018	6418	525 (8.2)	308 (4.8)	210 (3.3)	7 (0.1)
2019	5705	479 (8.4)	269 (4.7)	198 (3.5)	12 (0.2)
2020	5767	632 (11.0)	370 (6.4)	250 (4.3)	12 (0.2)
2021	6207	619 (10.0)	386 (6.2)	218 (3.5)	15 (0.2)
2022	5722	505 (8.8)	303 (5.3)	192 (3.4)	10 (0.2)

### Patient Characteristics

Characteristics of men presenting with a MRE within a population of all men presenting with metastatic prostate cancer are presented in Table 2. Older age was associated with higher risk of presenting with a MRE. For example, compared to the risk in men aged ≥80 years (9.8% [1604/16452]), the risks in men aged between 70and 79 years (8.0% [1470/18397]; aRR 0.82, 95% CI 0.77–0.88) and in men aged between 60 and 69 years (8.9% [916/10297]; aRR 0.91, 95% CI 0.84–0.98) were significantly lower.

**Table 2:** Associations between the characteristics of men presenting with primary metastatic prostate cancer and a MRE.

Variable	All primary metastatic prostate cancer, *N*	Metastatic presentation with a MRE		Metastatic presentation with a MRE aRR*	
		*N* (%)	χ^2^ *P*	aRR (95% CI)	*P*
Total	48 171	4272 (8.9)			
Age (years)			<0.001		<0.001
≥80	16 452	1604 (9.8)		1	
70–79	18 397	1470 (8.0)		0.82 (0.77–0.88)	
60–69	10 297	916 (8.9)		0.91 (0.84–0.98)	
18–59	3025	282 (9.3)		0.95 (0.84–1.07)	
Ethnicity			0.001		0.010
White	42 885	3781 (8.8)		1	
Black	1249	109 (8.8)		0.84 (0.69–1.02)	
Asian	744	88 (11.9)		1.22 (1.00–1.49)	
Mixed	181	17 (9.4)		0.99 (0.63–1.55)	
Other	468	61 (13.1)		1.29 (1.01–1.64)	
Missing	2644	216 (7.8)		0.90 (0.79–1.02)	
Quintiles of Index of Multiple Deprivation			<0.001		<0.001
1 (least deprived)	10 865	868 (8.0)		1	
2	10 932	895 (8.2)		1.03 (0.94–1.22)	
3	10 140	978 (9.6)		1.18 (1.08–1.28)	
4	8625	825 (9.6)		1.18 (1.08–1.29)	
5 (most deprived)	7609	706 (9.3)		1.27 (1.15–1.40)	
NHS region			<0.001		<0.001
North‐East	8655	761 (8.8)		1	
North‐West	6233	288 (4.6)		0.52 (0.46–0.60)	
Midlands	9631	851 (8.8)		1.02 (0.92–1.12)	
East England	5889	598 (10.2)		1.19 (1.07–1.32)	
London	3951	461 (11.7)		1.32 (1.17–1.48)	
South‐East	7748	621 (8.0)		0.96 (0.86–1.06)	
South‐West	6064	692 (11.4)		1.33 (1.20–1.47)	

MRE = either MUO or SRE or a combination of both.

* The aRR is adjusted for age, ethnicity, index of multiple deprivation, and NHS region.

The risk of presenting with a MRE varied according to ethnic background, with the highest risks observed in Asian men (11.9% [88/744]; aRR 1.22, 95% CI 1.00–1.49) and in men from the ‘Other’ category (13.1% [61/468]; aRR 1.24, 95% CI 1.01–1.64), compared to White men (8.8% [3781/42885]).

Men from more socioeconomically deprived neighbourhoods were more likely to present with a MRE. Men living in the most deprived national neighbourhood quintile had a risk of 9.3% (706/7609) of presenting with a MRE, compared to 8.0% (868/10865) in those living in the least deprived national neighbourhood quintile (aRR 1.27, 95% CI 1.15–1.40).

There was variation among the seven English NHS regions in the proportion of men presenting with a MRE at diagnosis, ranging from 4.6% (288/6233) in the North‐West of England to 11.7% (461/3951) in London. Compared to the North‐East (8.8%), the risk was significantly lower in the North‐West (aRR 0.52, 95% CI 0.46–0.60) and significantly higher in London (aRR 1.32, 95% CI 1.17–1.48) and the South‐West of England 11.4% (aRR 1.33, 95% CI 1.20–1.47).

Tumour characteristics were similar across the different presenting groups, except in men presenting with MUO, who had a greater proportion of T4 disease. Complete tumour characteristics, including PSA at diagnosis, Gleason score, T‐stage, and N‐stage, of the men who present with metastatic disease without a MRE, MUO, SRE, or a combination of MUO and SRE are presented in Data S1 Appendix 3.

### Route to Diagnosis

Of the 4272 men presenting with MREs, 2718 (63.6%) were diagnosed via an emergency pathway route. In contrast, only 8215 (18.7%) of the 43 899 men who presented without MREs were diagnosed via an emergency pathway route. The remaining men were diagnosed via urgent cancer or routine referrals.

### Mortality

Within our follow‐up period (5 years after diagnosis or up to 31 July 2024, whichever came earlier), 29 805 (61.9%) of the 48 171 men diagnosed with metastatic prostate cancer at the time of presentation had died, of whom 22 974 (77.1%) died from prostate cancer.

The median survival was 18 months for men presenting with MUO, 24 months for men presenting with a SRE, 10 months for men presenting with both MUO and a SRE, and 36 months for men presenting with metastatic disease without a MRE. With regards to different SRE subtypes, median survival for men presenting with the need for palliative radiotherapy alone was 26 months, compared to 24 months for spinal cord compression, and 21 months for pathological fracture with or without bone surgery.

**Fig. 2:**
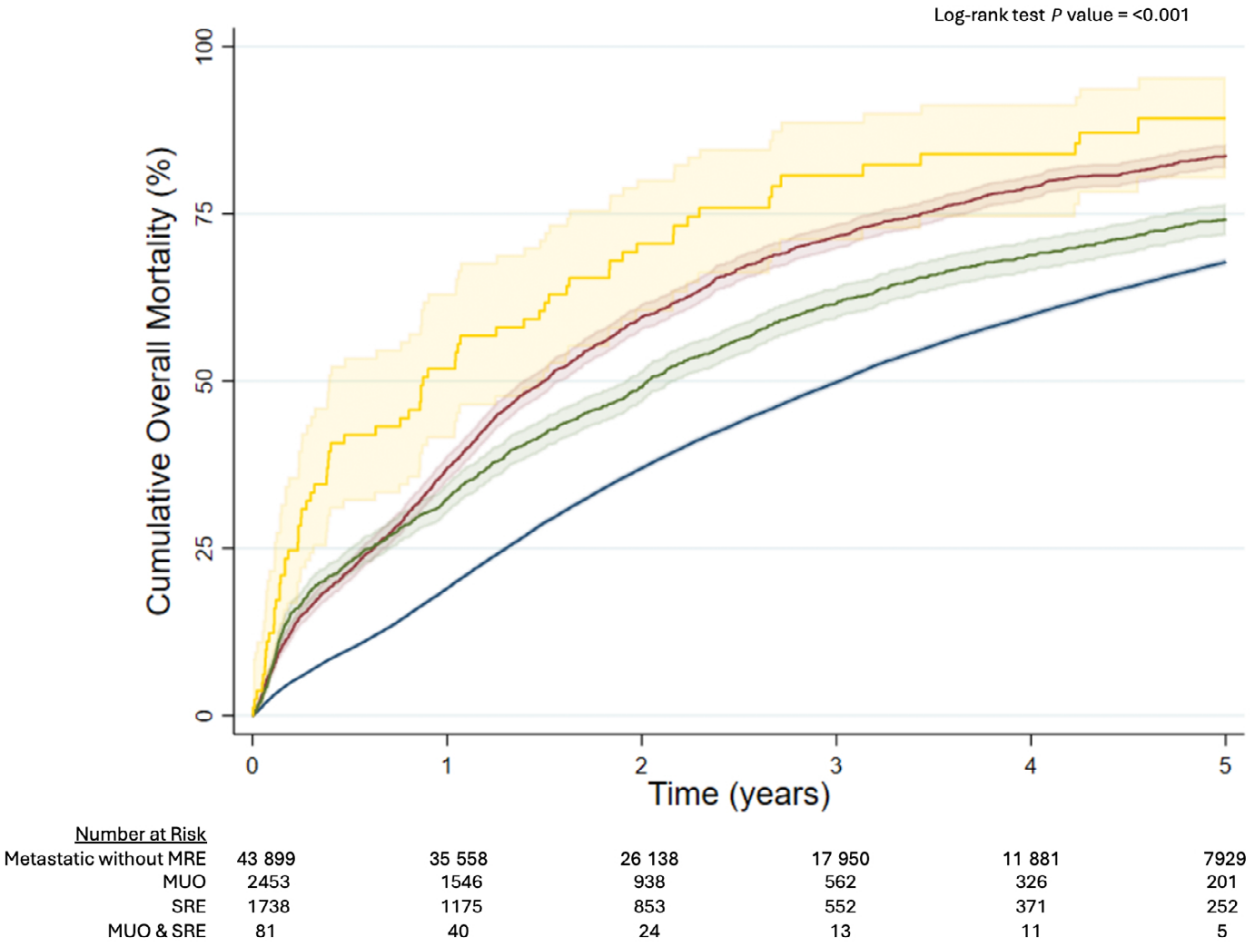
Cumulative overall mortality for men diagnosed with primary metastatic prostate cancer without a MRE (blue line), MUO (red line), a SRE (green line), or a combination of MUO and a SRE (yellow line).

**Fig. 3:**
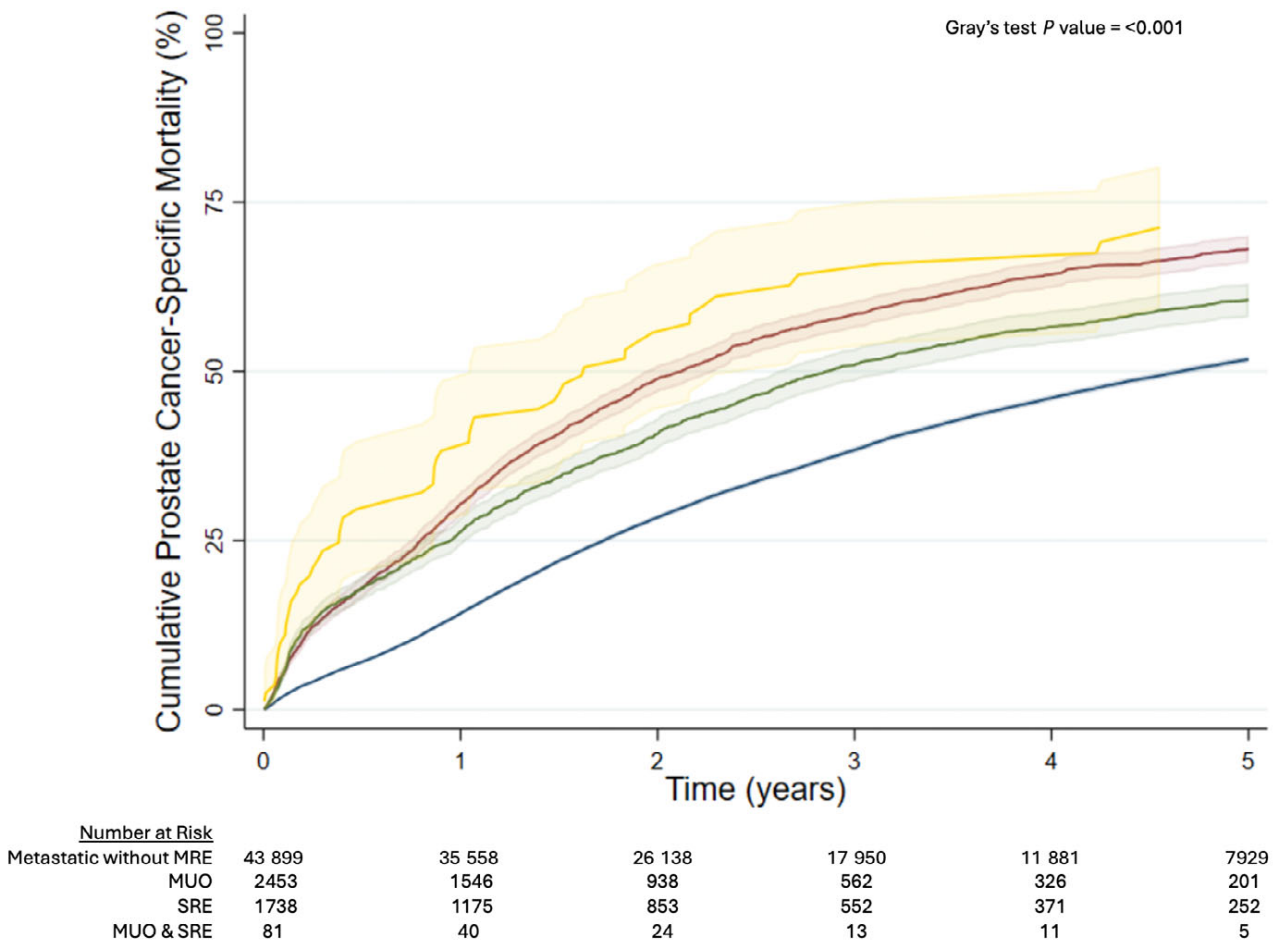
Cumulative prostate cancer‐specific mortality for men diagnosed with primary metastatic prostate cancer without a MRE (blue line), MUO (red line), a SRE (green line), or a combination of MUO and a SRE (yellow line).

For men diagnosed with metastatic prostate cancer with MUO at presentation, 1‐year overall mortality was 37.1% (95% CI 35.2–39.0%), 3‐year overall mortality was 71.7% (95% CI 69.8–73.5%), and 5‐year overall mortality was 83.8% (95% CI 82.1–85.5%). For men with a SRE at presentation 1‐year overall mortality was 32.5% (95% CI 30.3–34.7%), 3‐year overall mortality was 61.6% (95% CI 59.2–63.9%), and 5‐year overall mortality was 74.2% (95% CI 71.9–76.5%). For men with a combination of MUO and a SRE at presentation 1‐year overall mortality was 51.9% (95% CI 41.5–63.1%), 3‐year overall mortality was 80.7% (95% CI 71.1–88.8%), and 5‐year overall mortality was 89.3% (95% CI 80.3–95.4%). For men diagnosed with metastatic prostate cancer without a MRE at presentation, 1‐year overall mortality was 19.1% (95% CI 18.7–19.4%), 3‐year overall mortality was 49.9% (95% CI 49.4–50.3%), and 5‐year overall mortality was 67.8% (95% CI 67.3–68.3%) (*P* < 0.001) (Fig. 2). The results for prostate cancer‐specific mortality followed a similar pattern (Fig. 3).

## Discussion

To our knowledge, this is the first study to report the number of men who present with newly diagnosed metastatic prostate cancer and a concurrent MRE. Of those diagnosed with primary metastatic disease, nearly one in 10 (8.9%) presented with a MRE (5.1% with MUO, 3.6% with a SRE, and 0.2% with both). Older men and men living in more socioeconomically deprived neighbourhoods were more likely to present with a MRE. Those presenting with a MRE had worse survival outcomes.

During the 8‐year study period, the proportion of men presenting with a MRE at the time of their prostate cancer diagnosis remained stable, apart from a period during the COVID‐19 pandemic when the proportion increased. This rise is a likely consequence of the 31% drop in overall prostate cancer diagnoses following the national lockdown and reduced healthcare utilisation, causing a relative change in the balance between early and late‐stage presentation [23, 24]. This is likely to have affected men without symptoms of prostate cancer more than those with symptoms, resulting in a higher proportion of men presenting with symptomatic MREs. Ensuring availability of cancer diagnostic services and continuity of urgent referral pathways during periods of healthcare service pressure may prevent increases in men presenting with MREs in the future.

In a recently published study, we showed that men from more socioeconomically deprived neighbourhoods were more likely to present with metastatic prostate cancer. This study also demonstrated that neighbourhoods with a higher incidence of newly diagnosed metastatic prostate cancer had lower overall rates of prostate cancer diagnosed, likely due to differences in PSA testing [25]. Our study found similar patterns of presentation, with men from more socioeconomically deprived neighbourhoods more likely to present with a MRE and most men diagnosed with a MRE were diagnosed by presenting to their emergency department rather than via the outpatient cancer referral pathway. This suggests that their prostate cancer was only diagnosed after they developed signs or symptoms, raising concerns about their access to PSA testing [26, 27]. Targeted or risk‐stratified screening, particularly for men from more deprived neighbourhoods, certain ethnic groups and those with positive family history, may reduce the incidence of newly diagnosed metastatic disease and MREs at presentation [28]. Given the substantial morbidity associated with MREs, our results support further evaluation of early detection strategies. The population‐level incidence of MREs may serve as a meaningful indicator of the effectiveness of future screening programmes and other public health policy interventions.

We found that men from London were at a greater risk of presenting with a MRE compared to men from the North‐West of England. This is notable as previous population‐level evidence demonstrates a higher incidence of metastatic prostate cancer in the North‐West than in London [25]. This paradox may reflect a combination of factors. Although variation in coding practices may contribute, regional differences in referral, diagnostic and therapeutic practices are also plausible. The identification of MREs requires access to timely imaging (such as CT for MUO or MRI for spinal cord compression) as well as the availability and utilisation of therapeutic interventions, including decompression surgery or palliative radiotherapy. Finally, regional inequalities in access to primary care, thresholds for investigation and delays in referral may influence the stage at which men first present, leading to a higher likelihood of MREs in some regions [29].

Men who presented with a MRE had worse survival outcomes than men who presented without a MRE. These men experience the combined burden of their MRE and the underlying malignancy, often requiring a range of simultaneous treatments [30, 31]. Ensuring timely treatment of the complication as well as early investigation and management of the underlying cancer may help improve survival outcomes. However, to our knowledge, there is no published evidence about how men who present with a MRE are currently being treated, either for their cancer specifically or for the complications it engenders.

A limitation of our study is that staging data were missing for ~10% of patients, which may have led to an underestimation of the actual number of men diagnosed with metastatic disease in the English NHS, both with and without a MRE, by a similar proportion. Furthermore, in this descriptive study, the mortality outcomes were not adjusted for patient or tumour characteristics, which represents an area for detailed analysis in future work.

In conclusion, the risk of presenting with a MRE at the time of prostate cancer diagnosis varied significantly by age, socioeconomic deprivation, and residential region. Men presenting with a MRE at the time of diagnosis have worse survival outcomes and in particular, men with a combination of MUO and a SRE had a very poor prognosis. Further research is ongoing by our research group and others to better understand the nature of this problem, inform potential methods to avoid men presenting in this manner and suggest strategies to manage men if they do present in this way.

## Authors Contributions

Conception and design: Arjun Nathan, Matthew G. Parry, Joanna Dodkins, Ajay Aggarwal, Jan van der Meulen, James S. A. Green, Alison Tree, Noel Clarke, Thomas E. Cowling. Acquisition of data: Adrian Cook, Emily Mayne, Marina Parry.

Analysis and interpretation of data: Arjun Nathan, Matthew G. Parry, Adrian Cook, Emily Mayne, Raghav Varma, Jan van der Meulen, James S. A. Green, Alison Tree, Noel Clarke, Thomas E. Cowling. Drafting of the manuscript: Arjun Nathan, Ajay Aggarwal, Jan van der Meulen, James S. A. Green, Alison Tree, Noel Clarke, Thomas E. Cowling. Review of final manuscript: all authors.

## Ethical Approval and Consent

This study was conducted as part of the National Prostate Cancer Audit (NPCA), which has established regulatory approval, data security and governance procedures. Since this research involves anonymised secondary data, UK National Research Ethics Committee approval has not been sought per their guidelines. The study was performed per the Declaration of Helsinki.

## Disclosure of Interests

Alison Tree reports research grants from Accuray Incorporated, Elekta, Varian and Artera AI and honoraria from Astellas, Janssen, Elekta and Bayer. Noel Clarke reports funding from Astellas and Janssen.

## Funding

Arjun Nathan was funded by a National Institute for Health and Care Research (NIHR) Doctoral Research Fellowship (grant ref: NIHR304655). Arjun also acknowledges funding from The Urology Foundation and the Charles Reynolds Foundation. Alison Tree is supported by a Cancer Research UK Radiation Research Centre of Excellence at The Institute of Cancer Research and The Royal Marsden NHS Foundation Trust (grant ref: A28724 and RRCOER‐Jun24/100006) and a Cancer Research UK Programme Grant (ref: C33589/A28284). Alison also acknowledges NHS funding to the NIHR Biomedical Research Centre at The Royal Marsden and The Institute of Cancer Research. The views expressed in this publication are those of the author(s) and not necessarily those of the NHS, the National Institute for Health Research or the Department of Health and Social Care. This study was undertaken alongside the National Prostate Cancer Audit. The audit is commissioned by the Healthcare Quality Improvement Partnership (HQIP) as part of the National Clinical Audit and Patient Outcomes Programme, funded by NHS England and the Welsh Government (https://www.hqip.org.uk/national‐programmes). Neither HQIP nor NHS England had any involvement in the study design, in the collection, analysis, and interpretation of data, in the writing of the report, or in the decision to submit the article for publication.

## Supporting information


**Data S1.** Supporting Information.

## Data Availability

Data are available on reasonable request. Data are available from the Data Access Request Service, NHS England (https://digital.nhs.uk/services/data‐access‐request‐service‐dars). Statistical coding data are available upon reasonable request.
