# Supplementary material for: Characteristics and outcomes of men presenting with complications of metastatic prostate cancer
Source: BJU Int. 2026 Feb 17;137(5):840–8. doi: 10.1111/bju.70179 (PMC13071549; doi:10.1111/bju.70179)
Supplement: Supplementary file 1 — Data S1. Supporting Information. [file BJU-137-840-s001.docx]

**APPENDIX**

**Appendix 1.** International Classification of Diseases (ICD-10) Diagnostic Codes and Office of Population Censuses and Surveys (OPCS-4) procedure codes used to define malignant ureteric obstruction and skeletal-related events.

| **Malignant Ureteric Obstruction** | |
| --- | --- |
| N13.1 | Hydronephrosis with ureteric stricture, not elsewhere classified |
| N13.3 | Other and unspecified hydronephrosis |
| N13.4 | Hydroureter |
| N13.5 | Kinking and stricture of ureter without hydronephrosis |
| N13.6 | Pyonephrosis (Hydroureternephrosis with infection) |
| N13.8 | Other obstructive and reflux uropathy |
| N13.9 | Obstructive and reflux uropathy, unspecified |
| *N13.0*  *N13.2* | **Excluded Diagnoses**  *Hydronephrosis with PUJ Obstruction*  *Hydronephrosis with renal and ureteral calculous obstruction* |
| **Skeletal Related Events** | |
| C795 | **Bone Metastasis** |
|  | **Pathological Fracture** |
| M48.5 | Collapsed vertebra, not elsewhere classified |
| M49.5 | Collapsed vertebra, due to metastasis |
| M84.4 | Pathological fracture, not elsewhere classified |
| M90.7 | Fracture of bone in neoplastic disease |
|  | **Spinal Cord Compression** |
| G55.0 | Nerve root and plexus compressions in neoplastic disease |
| G83.4 | Cauda equina syndrome |
| G95.2 | Cord compression |
| G95.8 | Other specified diseases of spinal cord |
| G95.9 | Disease of spinal cord, unspecified |
| G99.2 | Myelopathy in diseases classified elsewhere |
|  | **Bone or Spinal Surgery** |
| V22-27, V67-68 | Decompression operations on spine |
| V28 | Insertion of lumbar interspinous process spacer |
| V38 | Fusion of joint/stabilisation of spine |
| V41 | Instrumental correction of deformity of spine |
| V43, V47 | Extirpation of lesion/biopsy of spine |
| V44-46 | Decompression/reduction/fixation of fracture of spine |
| V55 | Levels of spine |
| W05 | Prosthetic replacement of bone |
| W08-09 | Excision of bone/extirpation of lesion of bone |
| W16 | Division of bone |
| W19-26 | Reduction of fracture |
| W28, W30 | Internal/external fixation of bone |
| W37-41, W93-95 | Replacement of hip/knee joint |
| W46-48 | Prosthetic replacement of head of femur |
| W65-67 | Reduction of traumatic dislocation of joint |

**Appendix 2.** Histogram showing the percentage of men diagnosed at various time intervals between the date of diagnosis and the date of the MRE (MUO or a SRE) with a corresponding model of cumulative incidence of mortality between the different definitions of a MRE ‘at presentation’.

**
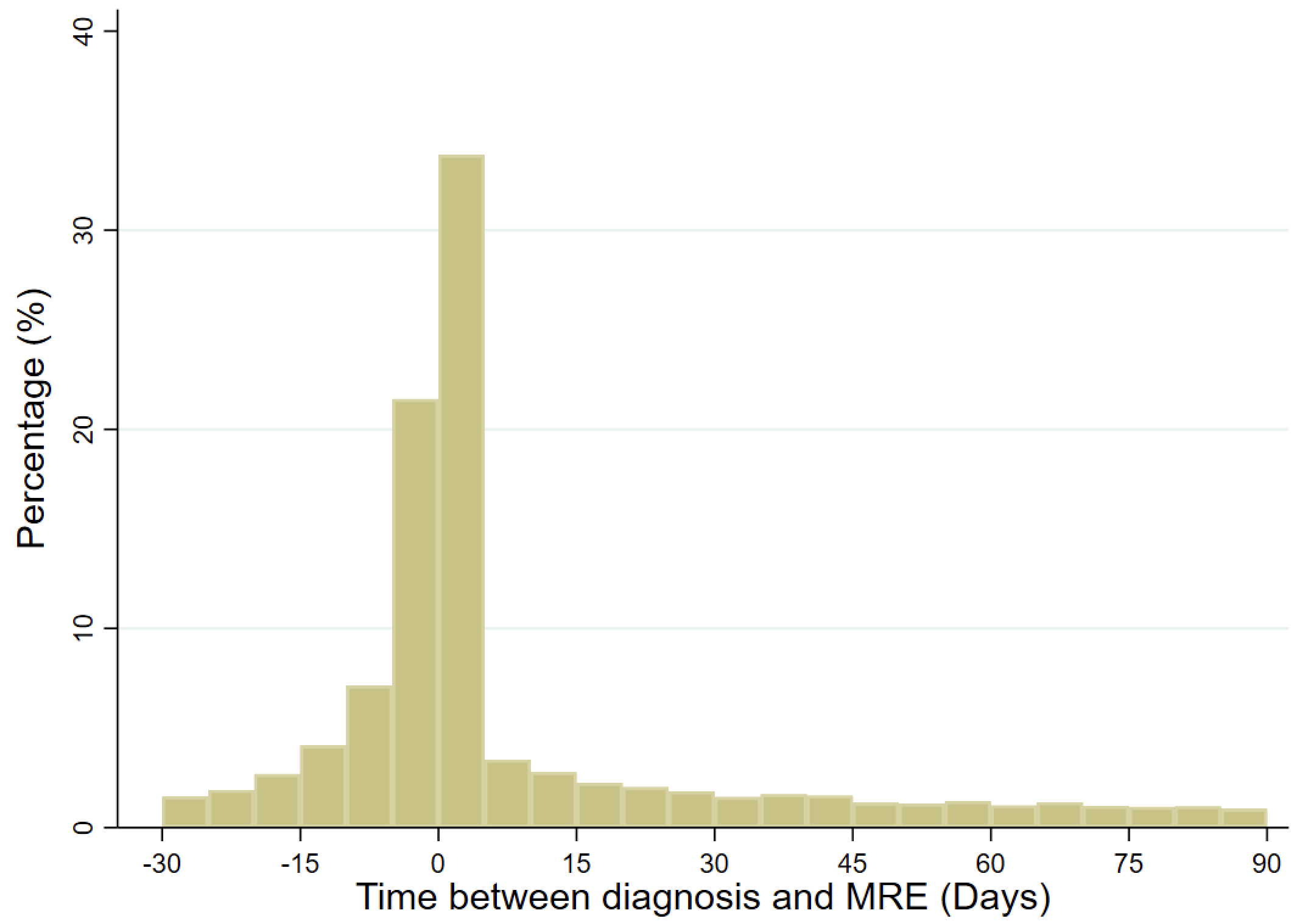
**

**Appendix 3. Tumour characteristics of men presenting with primary metastatic prostate cancer and different metastatic-related events (MREs), including malignant ureteric obstruction (MUO) and skeletal-related events (SREs)**

|  | **Metastatic only** | | **Presenting with a MRE** | | | | | |
| --- | --- | --- | --- | --- | --- | --- | --- | --- |
|  |  |  | **MUO** | | **SRE** | | **MUO and SRE** | |
| **All** | **n** | **%** | **n** | **%** | **n** | **%** | **n** | **%** |
|  | 43,899 |  | 2,453 |  | 1,738 |  | 81 |  |
| **PSA** |  |  |  |  |  |  |  |  |
| <10 | 1,966 | *4.5* | 68 | *2.8* | 29 | *1.2* | 1 | *1.2* |
| 10-20 | 2,348 | *5.3* | 67 | *2.7* | 22 | *0.9* | 0 | *0.0* |
| >20 | 22,908 | *52.2* | 1,162 | *47.4* | 791 | *32.2* | 37 | *45.7* |
| Missing | 16,677 | *38.0* | 1,156 | *47.1* | 896 | *36.5* | 43 | *53.1* |
| **Gleason score** |  |  |  |  |  |  |  |  |
| 6 | 280 | *0.6* | 7 | *0.3* | 5 | *0.2* | 0 | *0.0* |
| 7 | 3,883 | *8.8* | 80 | *3.3* | 48 | *2.0* | 1 | *1.2* |
| 8 | 4,350 | *9.9* | 123 | *5.0* | 85 | *3.5* | 3 | *3.7* |
| 9 | 12,221 | *27.8* | 561 | *22.9* | 196 | *8.0* | 14 | *17.3* |
| 10 | 1,491 | *3.4* | 135 | *5.5* | 25 | *1.0* | 1 | *1.2* |
| Missing | 21,674 | *49.4* | 1,547 | *63.1* | 1,379 | *56.2* | 62 | *76.5* |
| **T stage** |  |  |  |  |  |  |  |  |
| 1 | 598 | *1.4* | 37 | *1.5* | 19 | *0.8* | 0 | *0.0* |
| 2 | 2,779 | *6.3* | 70 | *2.9* | 48 | *2.0* | 2 | *2.5* |
| 3 | 15,927 | *36.3* | 515 | *21.0* | 312 | *12.7* | 6 | *7.4* |
| 4 | 8,268 | *18.8* | 679 | *27.7* | 212 | *8.6* | 18 | *22.2* |
| Missing | 16,327 | *37.2* | 1,152 | *47.0* | 1,147 | *46.8* | 55 | *67.9* |
| **N stage** |  |  |  |  |  |  |  |  |
| 0 | 10,171 | *23.2* | 335 | *13.7* | 216 | *8.8* | 4 | *4.9* |
| 1 | 16,326 | *37.2* | 994 | *40.5* | 459 | *18.7* | 26 | *32.1* |
| Missing | 17,402 | *39.6* | 1,124 | *45.8* | 1,063 | *43.3* | 51 | *63.0* |
